# A multi-level community-based systems dynamic approach to advance equitable adolescent sexual and reproductive health in Chicago

**DOI:** 10.3389/fpubh.2026.1857998

**Published:** 2026-07-30

**Authors:** Whitney R. Garney, Kelly Wilson, Meagan Miller, Christi Esquivel, Kobi V. Ajayi

**Affiliations:** 1 Center for Community Health and Aging, College Station, TX, United States; 2 Department of Health Behavior, School of Public Health, Texas A&M University, College Station, TX, United States; 3 College of Nursing, Texas A&M University, Bryan, TX, United States

**Keywords:** adolescents, community-based system dynamics, evidence-based programs, health equity, sexual and reproductive health, system thinking

## Abstract

**Background:**

Teenage pregnancy in the United States (U.S) continues to be a serious public health concern, despite the significant declines in birth rates documented over the last three decades. Risk factors leading to teen pregnancy go far beyond individual-level behavior, like engaging in risky sexual behaviors, and can be linked to a web of interconnected ecological influences outside of an adolescent’s proximal locus of control. Individual behaviors, interpersonal relationships, community environments, and structural conditions interact in complex ways to shape adolescents’ sexual and reproductive health (SRH) outcomes. These multi-level influences highlight the need for a systems approach capable of capturing the complexity of teen pregnancy and its associated risk factors. The Chicago Partnerships & Opportunities for Adolescent Health Preparation, Support & Services (POPSS) is a multi-level initiative that aims to improve adolescent sexual and reproductive health, support positive youth development, and advance health equity by expanding the use of medically accurate, age-appropriate, evidence-based teen pregnancy prevention programs. In this report, we describe the POPSS project, which addresses multi-level factors contributing to teen pregnancy in Chicagoland through a systems SRH initiative.

**Context:**

This project used community-based systems dynamics (CBSD) to conceptualize the challenges of teenage pregnancy. This was conducted through community listening sessions, annual iterative systems dynamic mapping, and continuous improvement integrated throughout the project to rapidly respond to changes. With an understanding of the system’s complexity, the POPSS stakeholders identified diverse implementation settings, such as healthcare, schools, and community settings where adolescents congregate, and evidence-based teen pregnancy prevention programs to ultimately enhance adolescent SRH equity.

**Conclusion:**

Increasing equitable access to evidence-based teen pregnancy prevention programs hinges on a systems approach operationalized through strategic partnerships.

## Introduction

Teenage pregnancy in the United States (U.S) continues to be a serious public health issue, despite the significant declines in birth rates documented over the last three decades that have resulted from increased access to birth control, public health interventions, education, and policy changes (1–5). Currently, the U.S. has a higher teen birth rate than most other high-income nations: 14 births per 1,000 females in 2021, compared with 9.2 per 1,000 in Australia and 2 per 1,000 in Switzerland (1, 6, 7). Teen pregnancies carry substantial social, economic, and health implications for adolescents, their families, and societies (8). Teen parents often face interrupted education, limited employment opportunities, and increased risk of poverty, while their children are more likely to experience adverse developmental outcomes (1, 8–10). However, teen pregnancy differs across racial, socioeconomic, and geographic lines (8, 11). Females from non-Hispanic American Indian/Alaska Native (24), non-Hispanic Native Hawaiian and other Pacific Islander (22), non-Hispanic Black (22), and Hispanic backgrounds (21) have persistently higher rates than non-Hispanic White and Asian females (1). Furthermore, adolescents experiencing poverty, food, housing, and transportation insecurities, limited access to healthcare, or unstable family environments have disproportionately higher rates (8, 10, 12, 13). The rates also differ by states and regions in the U.S. (14, 15). These patterns underscore that teen pregnancy cannot be viewed through an individual behavioral lens; risk emerges from a web of interconnected, ecological influences. Individual behaviors, interpersonal relationships, community environments, and structural conditions interact in complex ways to shape adolescents’ sexual and reproductive health (SRH) outcomes. Understanding these multi-level factors highlights the need for a holistic approach capable of capturing the complexity of the multi-level systems that contribute to teen pregnancy.

Systems approaches offer a unique lens to examine adolescent SRH because it moves beyond traditional, linear explanations of health behaviors (16). Rather than isolating individual risk factors, systems thinking emphasizes the interconnectedness of the environments in which adolescents live, learn, and make decisions (17, 18). In the context of adolescent SRH, systems approaches suggest that adolescents are embedded within overlapping systems, including families, peer networks, schools, healthcare institutions, neighborhoods, and policy environments, that influence their knowledge, attitudes, and behaviors related to SRH (16, 17, 19). A systems approach allows stakeholders to consider how these systems interact, how feedback reinforces certain behaviors, and how changes in one part of the system can produce ripple effects across the rest of the system (19). For example, improving access to contraception may have a limited impact if adolescents face stigma when seeking reproductive healthcare or if school environments do not provide comprehensive SRH education (21, 20). Additionally, a school’s disciplinary policy might lead to higher dropout rates, which in turn increases the likelihood of early parenthood (22). By mapping how teenagers’ reproductive choices are influenced by feedback loops, systems thinking allows for a holistic approach to addressing teen pregnancies.

Applying systems thinking to adolescent SRH offers several important benefits. First, it enables a deeper exploration of the root causes of teen pregnancy by identifying the proximal drivers and the structural and contextual forces that shape adolescent risk behaviors (18, 19). This broader perspective helps uncover leverage points (i.e., places within the system where targeted interventions can produce the most beneficial, meaningful, and sustained change). Furthermore, a systems approach supports the development of multi-level interventions that address several determinants simultaneously. For instance, a systems approach might combine school-based SRH education, youth empowerment activities, community health services, and policy initiatives to reduce socioeconomic barriers (18). Systems thinking requires interdisciplinary and multisectoral partnerships. Because teen pregnancy is influenced by diverse factors, stakeholders in education, healthcare, social services, and community organizations must work together rather than operate in isolation. Finally, systems approaches uniquely contribute to fostering the capacity to capture feedback dynamics, unintended consequences, and nonlinear relationships, enhancing comprehensive evaluation by allowing stakeholders to examine how interventions influence behaviors over time (23). This is particularly valuable in adolescent SRH, where shifts in norms, relationships, and community conditions may take time to manifest in measurable changes.

Despite its advantages, several barriers limit the widespread application of systems thinking in addressing teen pregnancy. One major challenge is the complexity of systems-level projects, which require interdisciplinary expertise, local data, and analytical tools that many organizations may not possess (24, 25). Additionally, systems approaches can be difficult to translate into policy, as policymakers often prefer straightforward solutions and immediate results (24). Fragmentation across service sectors in the U.S. also poses a barrier; healthcare providers, schools, and social service agencies frequently operate independently, making it difficult to coordinate multi-level community interventions (26). Cultural, religious, and political resistance to comprehensive SRH education further complicates efforts to implement systems-informed strategies (3, 27). For example, comprehensive education varies drastically across K-12 institutions in the U.S., with some states focusing on abstinence-only education, thereby limiting the reach of institutions or types of programming addressing teen pregnancy (28). These barriers highlight the need for research that clarifies how systems approaches can be operationalized in real-world settings and demonstrates their value in improving adolescent SRH outcomes. Projects grounded in systems approaches can help address these gaps by offering evidence-based insights into how multi-level factors interact and how interventions can be designed to address them more effectively.

The purpose of this study is to describe a systems approach to address multilevel factors contributing to teen pregnancy in one U.S. state. The significance of this work lies in its potential to understand proximal mechanisms, such as individual-level risks through which system-level dynamics frequently operate, to a broader system-oriented perspective that acknowledges the complexity of adolescent lives. These shifts ensure that interventions are more responsive, equitable, and sustainable. Ultimately, we seek to contribute to the growing body of literature advocating for systems approaches in public health.

## Context

### The Chicago partnerships and opportunities for adolescent health preparation, support and services (POPSS) initiative program description

In 2023, Texas A&M Health was awarded a five-year grant from the U.S. Department of Health and Human Services (HHS) as part of the Teen Pregnancy Prevention program (TPP) to implement the Chicago Partnerships & Opportunities for Adolescent Health Preparation, Support & Services (POPSS) initiative, in collaboration with Ann & Robert H. Lurie Children’s Hospital of Chicago. Per HHS, the initiative aims to improve adolescent sexual and reproductive health, support positive youth development, and advance health equity by expanding the use of medically accurate, age-appropriate, evidence-based teen pregnancy prevention programs (EBPs) (29). EBPs are programs that have been rigorously evaluated and shown to reduce teen pregnancy and related risk behaviors (30).

The POPSS Project is a multi-level community-based systems project that focuses on programming and supports within 3 unique settings: health clinics, schools, and community settings. It utilizes a multi-sector advisory board to plan and implement programming across its catchment area. Using a team of health educators, the project concurrently implements EBPs, trains stakeholders in adolescent health topics, conducts outreach, and promotes adolescent-friendly health clinics to its constituents.

#### Setting

POPSS works across communities in Chicagoland due to disparities in adolescent sexual and reproductive health outcomes, as well as adverse social determinants of health. The 2019 Youth Risk Behavior Surveillance System (YRBSS) indicated high school youth in Chicago, IL, were significantly more likely than youth across the US to have sex without using condoms or a form of hormonal birth control (31). More so, in 2020, Chicago, IL, experienced a teen birth rate of 14.1 per 1,000 females ages 15–19 (31). While the rates have declined to 12.3 per 1,000 females ages 15–19 in 2023, Black and Hispanic teens (15.6 each) have even higher rates than the state average (32). There continue to be disparities for certain racial/ethnic groups, most notably within Hispanic and African American communities. Furthermore, sexually transmitted infections (STIs) are also of concern as data demonstrates rates of chlamydia and gonorrhea in Chicago are on the rise, and have been for years, with most cases diagnosed among people ages 15–24 (31, 32)Data from 2021 indicate that only 1.7% of sexually active high school youth in Chicago, IL, reported being tested for an STI (other than HIV) in the past 12 months. In the selected county, over 10% of persons living in the county are without health insurance and living in poverty, increasing the access gap to sexual health education, resources, and services (31, 32).

### Environmental scan using community listening sessions

At the onset of the POPSS project, informal community listening sessions were used to gather early insights. Community listening sessions are strategic tools used in community-based research that can promote social learning by creating a structure that facilitates inclusive dialogue and reflection on the issue at hand (33). Overall, the listening sessions aimed to (1) convene the community & build relationships, (2) identify EBPs based on fit and cultural relevance, using a list of funder approved programs (3) engage stakeholders and network with partners, and (4) gather insight into the community’s perspective and relate priorities. Discussions focused on gathering input on the selected EBP, allowing community members to express support, concerns, or other feedback prior to implementation.

Community stakeholders were recruited through the Lurie Children’s Hospital Sexual Education Program’s existing network, and participants in the sessions included a mix of organizations that provide health and education services to youth in diverse settings. Because of the informal information gathering process, no participant demographics were collected. A total of 5 listening sessions were conducted. Listening sessions engaged strategic stakeholder groups including (1) congregant care staff from local transitional housing, (2) school administrators and teachers, (3) a youth advisory group, (4) healthcare workers from local community clinics, and (5) parents and caregivers. The sessions were led by the POPSS team and lasted approximately 45–50 min each.

### Approach: community-based systems dynamics

Following community listening sessions, the POPSS team used community-based systems dynamics (CBSD) to map complex interactions contributing to teenage pregnancy in Chicagoland with the goal of understanding the primary drivers of teenage pregnancy in their community (35). CBSD is an approach that helps communities understand complex problems and design solutions by involving the people who are directly affected by mapping and analyzing the systems around them (35). It blends systems thinking with participatory methods, enabling community members, in collaboration with experts, to become the drivers of insight and change. CBSD uses systems thinking tools to create a systems dynamic map that illustrates the interconnectedness of multi-level factors, feedback between factors, emergent properties, and time delays. These methods allow communities to understand the root causes of complex issues, visualize how different factors interact over time, understand structural drivers contributing to a problem, identify leverage points for interventions with the greatest impact, and co-create strategies that reflect the lived experience and local context (16, 35).

### CBSD activities

To conduct CBSD, an introductory session was held with community members and the POPSS project team to train them in the basics of systems and system-mapping processes. Topics covered included an introduction to community-based group modeling, causal loops, brainstorming processes, and affinity clustering. During the session, participants were presented with a hypothetical scenario to apply the skills they had learned in real time before the systems mapping sessions began.

**Figure 1:**
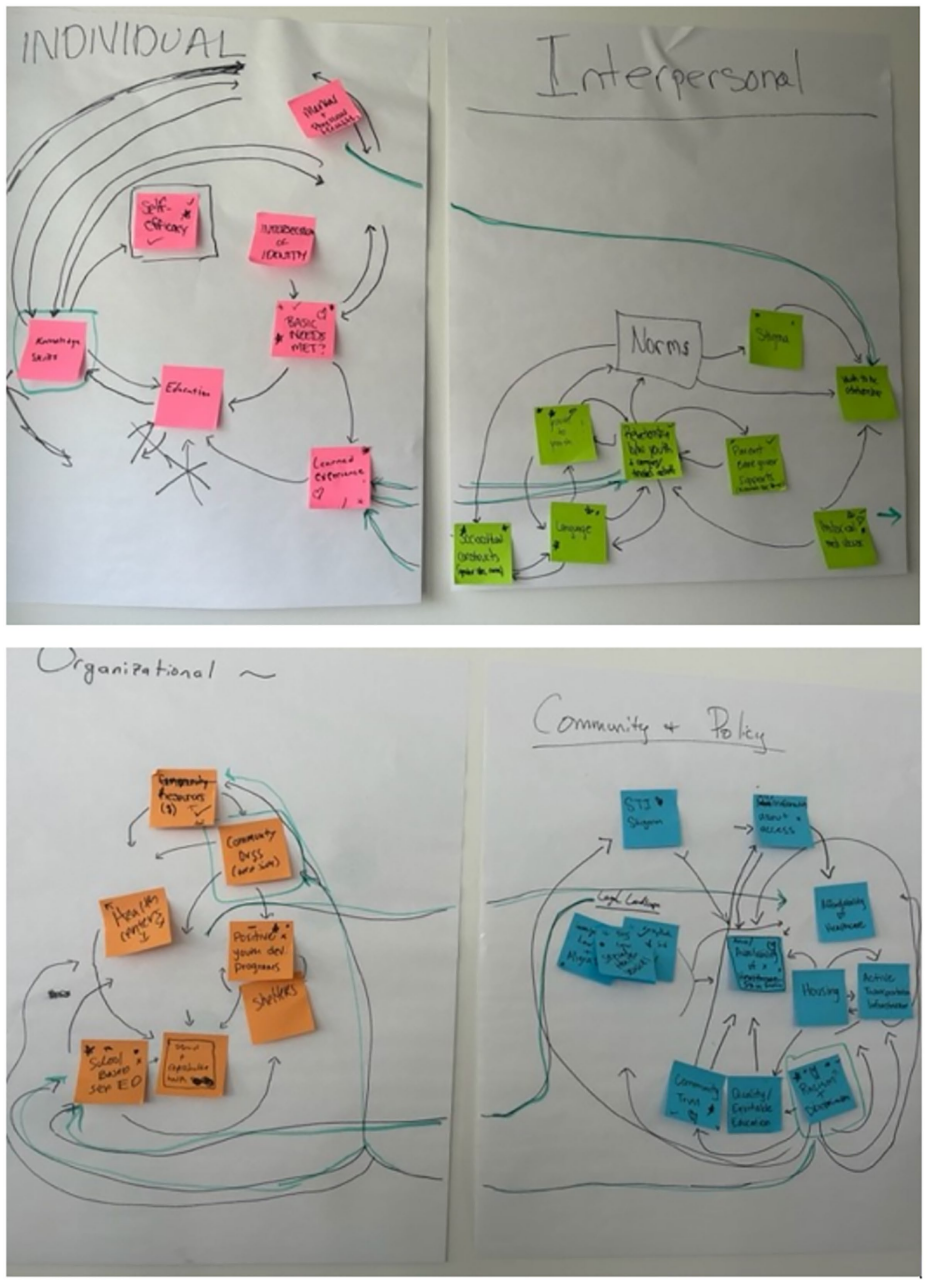
Example causal loop diagrams using socioecological levels.

Following this introductory session, the POPSS team organized a 3-h in-person system mapping session with 39 participants representing five communities. Participants were presented with data from the listening sessions and existing needs assessments. They engaged in an interactive mapping activity involving post-it notes, highlighters and markers, boards, and flashcards to build initial community dynamics maps. Facilitators used the levels of the socioecological model (9) (i.e., policy, community, interpersonal, and individual) to guide discussions and prompts, which helped participants consider diverse factors affecting adolescent sexual and reproductive health in Chicago. Participants developed 4 unique systems dynamics maps for each level of the socioecological model, as seen in Figure 1. The goal of each map was to illustrate the interconnected factors contributing to teenage pregnancy in Chicagoland, as they pertained to the respective level of analysis. By focusing on each level independently, participants ensured that more distal, indirect influences on teenage pregnancy were adequately conceptualized. After this session, the POPSS project team combined all four maps into one systems dynamics map.

**Figure 2:**
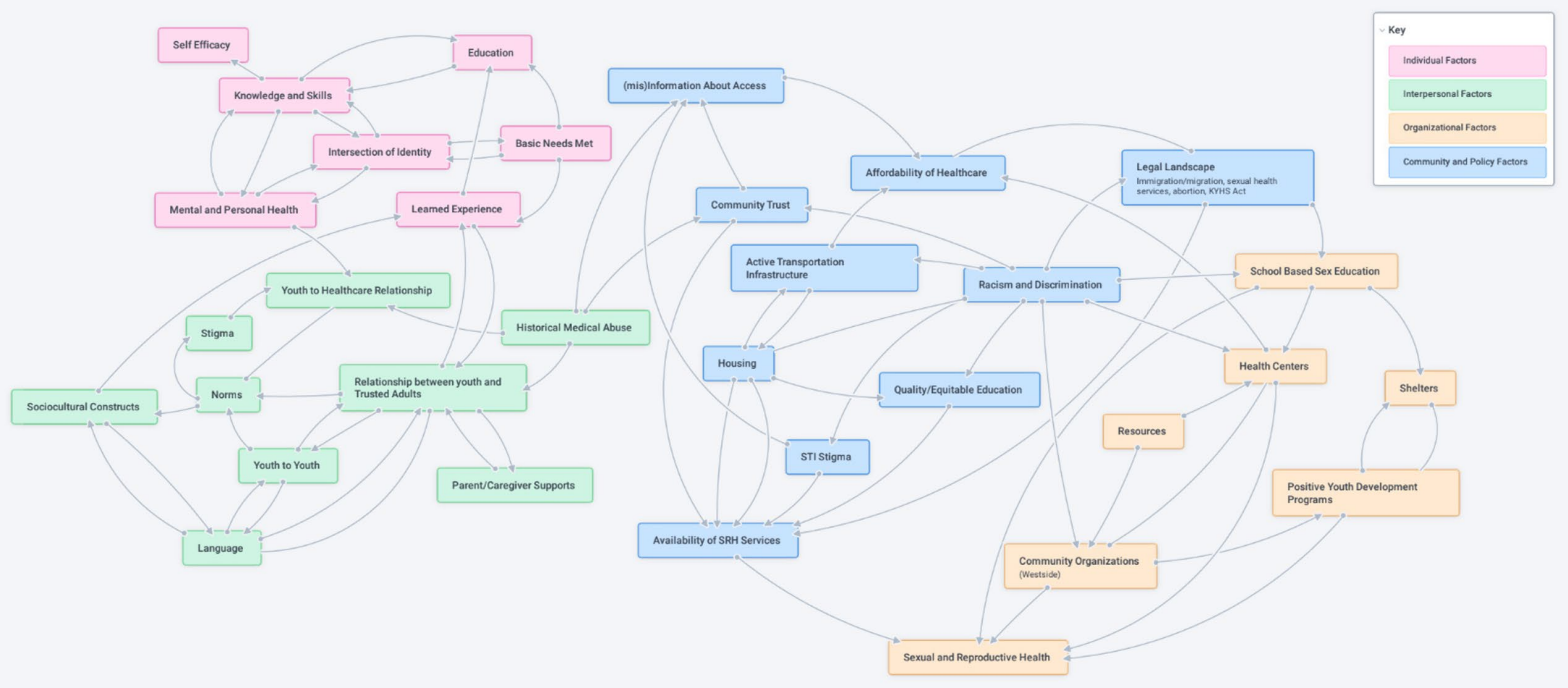
Year 1 system map based on the socioecological levels showing the causal loops across systems.

A 3rd virtual session was convened to present the combined systems dynamics model back to stakeholders. To begin validation, a tracing technique was used, and changes were made in real time. Then, stakeholders were tasked with sharing the 2nd iteration of the systems dynamics map with community partners to gain additional feedback. Over the next month, these partners suggested additional changes, which produced a 3rd iteration of the systems map. Lastly, the POPSS team presented a final version of the systems dynamics map to the project’s Steering Committee for final review. The final systems map developed in year 1 is reported in Figure 2.

The final year one map identified system drivers, which stakeholder identified as having a great potential impact on adolescent SRH in Chicago. The drivers included: (1) Knowledge and skills, (2) Relationship between youth and trusted adults, (3) Healthcare, and (4) Racism and Discrimination. These components were taken into consideration and directly impacted the identification of evidence-based programs (EBPs) for the community interventions. EBPs are described in detail below, however, each program was selected for its ability to address factors contributing directly to drivers 1–3. Driver 4, racism and discrimination, was determined to be a key component of all programs. Therefore, only EBPs that adequately accounted for inclusivity and trauma were considered. The final systems map developed in year 1 is reported in Figure 2.

Since the POPSS project is now entering its 4th funding year, the team made annual edits to the map in years 2 and 3 to reflect changes in community context and project priorities. Modifications were presented to community partners at project Steering Committee meetings each year to ensure edits were accurate and useful. Annual iterations of the map are particularly important for capturing real-time changes within the community and for ongoing evaluation. For example, at the end of year 2 (spring 2025), a series of executive orders were passed that substantially impacted the project. One of the original EPBs was replaced, a change that required significant time, reflection, and priority adjustments. As a result, the system’s map changed to illustrate an increased impact of policy and political climate on the project.

### Multi-level community-based intervention strategies

Based on the systems map and funder requirements, the POPSS project team chose three EBPs to implement in three unique settings: FLASH in school settings, Plan A in clinic settings, and LiFT in community settings (36, 37). At the outset, the POPSS project implemented IN*Clued in community settings, but has since been discontinued and replaced with LiFT (38). Each program and setting is described below.

*FLASH* is a nationally recognized, evidence-based sexual health education curriculum designed for students in grades 9–12. It consists of 15 lessons that can be delivered in either 15 or 10 sessions, offering flexibility in implementation in school settings. FLASH curriculum is widely used and rigorously evaluated, with the primary goals of preventing teen pregnancy, reducing the incidence of sexually transmitted diseases (STDs), and preventing sexual violence. Additionally, it aims to increase students’ knowledge of the reproductive system, puberty, and healthy relationships. These goals align specifically with the 1st driver in the systems map, knowledge and skills.

*Plan A* is a clinic-based intervention that uses a brief, engaging 23-min video. The video uses entertainment-education strategies to help reduce unplanned pregnancies and sexually transmitted infections (STIs) among young people. It is considered an evidence-based program, having demonstrated effectiveness through rigorous evaluations in reducing behaviors and risk factors associated with teenage pregnancy. Plan A facilitates healthcare access and addresses the 3rd structural driver, healthcare.

*LiFT* (Linking Families and Teens) is a nationally recognized, evidence-based sexual health education workshop designed for adolescents ages 13–19 and their parents or guardians. This widely used and rigorously evaluated program focuses on strengthening communication between teens and supportive adults, encouraging youth to open up while equipping parents and caregivers to guide teens in making healthy choices and achieving their goals. LiFT also fosters connections among parenting adults within the community. The program aims to prevent teen pregnancy, reduce the incidence of sexually transmitted diseases (STDs), and increase knowledge of the reproductive system, puberty, and healthy relationships. Designed as a family-centered workshop, LiFT is delivered over 6 h—either in one session or split into two three-hour sessions—and requires each participating teen to attend with at least one supportive adult. LiFT focuses on skill development related to the 2nd systems driver, relationships between youth and trusted adults.

While targeting unique settings and audiences, all three programs have reinforcing messaging. They also have linkages across settings; for example, families attending a LiFT workshop can be referred to youth-friendly clinics that implement Plan A. The 3-setting strategy ensures that messaging and education are shared with adolescents from multiple perspectives and sources. Furthermore, the POPSS steering committee includes representatives from implementing organizations, enabling it to leverage and expand program reach as new opportunities emerge. Table 1 shows the number of schools implementing FLASH, Plan A, and LiFt since POPSS inception.

**Table 1:** POPSS EBP implementation sites from YR 1–3.

FLASH	Plan A	LiFT
19 Schools	7 clinics	3 community organization

### Program evaluation

The POPSS initiative tracks implementation and system-level progress using performance measures established by the funder. These measures capture changes in program reach, partner engagement, dissemination capacity, and service linkage over time. Table 2 summarizes key indicators collected during the first 2 years of implementation.

**Table 2:** Community impact key indicators.

Performance measures	Description	Year #1 output	Year #2 output
Dissemination	Number of unique communication channels/medium the tPOPSS project uses to share information about teen pregnancy prevention and the TPP-funded grant project?	7	14
Partners	# of partners involved in the implementation of the project	41 partners	90 partners
Steering committee	# of steering committee members	11	18
Training	Professional development activities or technical assistance relevant to project implementation	91	132
Referrals	# of program participants referred by project staff to supportive services providers	15	35

Across this period, findings demonstrate substantial growth in system engagement and program infrastructure. The number of active partners more than doubled, reflecting the expansion of the multi-sector network and increased reach across community settings. Similarly, the number of dissemination channels increased, indicating an enhanced capacity to share information and promote adolescent sexual and reproductive health (SRH) resources across the service area. Increases in training and technical assistance activities further suggest strengthening of workforce capacity to deliver evidence-based programming.

Service linkage outcomes also showed early signs of partner activation as indicated by an increase in referrals between implementing organizations, While these increases are modest, they represent important early indicators of improved connectivity across sectors and potential pathways to care for adolescents.

Taken together, these early outcomes suggest that the POPSS initiative is contributing to the development of a more connected and responsive system, with gains in partnership development, dissemination, and implementation capacity. However, continued monitoring is needed to assess how these system-level changes translate into sustained program delivery and longer-term health outcomes.

### Continuous quality improvement

A continuous quality improvement (CQI) process was integrated into the evaluation to ensure the project is responsive to challenges in a timely manner. CQI activities are conducted through regularly scheduled project meetings and guided by the Plan–Do–Study–Act (PDSA) framework, an iterative four-step model used to identify, test, refine, and optimize implementation strategies over time. Within this framework, the project team systematically planned modifications, implemented changes, assessed outcomes, and used findings to inform subsequent cycles (39).

CQI data includes facilitator logs, program observations, organizational partner input, and network data, A PDSA cycle is implemented each 6 months to aggregate and examine data. Each cycle, a challenge has been identified. Importantly, CQI functions not only as a monitoring tool but as a mechanism for adaptive system management, allowing the initiative to respond to evolving community conditions and organizational dynamics. Key challenges and corresponding adaptations have included: (1) updating expected participation goals and reach estimates, (2) the replacement of one evidence-based program in Year 2 to better align with funder priorities; (3) modification of implementation settings to reflect shifting organizational capacity and participation; and (4) increasing participation in clinic programming.;

## Discussion

This study contributes to the growing body of literature examining how systems approaches can support the implementation of multi-level community-based public health interventions. Specifically, it adds to the literature by demonstrating how community-based systems dynamics (CBSD) approaches can be operationalized within adolescent SRH initiatives. Through the engagement of multi-sectoral partners, leveraging of existing community infrastructure, promotion of shared ownership, and iterative adaptation in response to evolving system conditions, the POPSS initiative provides a practical example of system thinking in action within a community health context. Collectively, these findings offer insight into how systems-oriented approaches may support implementation, collaboration, and sustainability in large-scale public health efforts.

### Multi-level, systems approach

In many community-based projects, interventions are conducted in multiple settings to target unique, but reinforcing risk factors. For example, a media campaign focused on community awareness of chlamydia and expanded clinic hours to increase STI testing. However, aside from information sharing between implementing organizations, there is typically no true integration like joint planning, shared resources, shared data and reporting, or staff crossover. Because the POPSS project uses a systems approach, the three unique program settings are more than multi-level change mechanisms, each setting operates with a shared perspective and metrics, referrals, staffing, and resources are optimized to programming. This approach was established using systems mapping, but has continued during implementation because of a common core team that works within and across settings. This allows each organization to receive optimal benefits with minimal burden. It reduces redundancies and leverages individual program success to make progress in other areas. For example, Plan A is implemented in an adolescent health clinic that serves pregnancy and parenting people. The clinic plans to implement a LiFT workshop that will teach young people and trusted adults how to have difficult conversations. At the same time, some schools implement both FLASH and Plan A to increase young people’s knowledge about risky behavior and provide STI testing and contraceptive access onsite through School Based Health Clinics. This type of overlap is only possible because of the systems approach, because it views the whole project as more than just the sum of each individual part. CBSD approaches were particularly useful in considering both independent and interconnected relationships between settings during the development and implementation of adolescent SRH interventions.

### Collaboration and shared ownership in complex systems

A critical component to understanding the complexities of SRH within Chicago was the engagement of diverse stakeholders through listening sessions and systems mapping activities. These processes helped ensure that intervention strategies were grounded in local context and met community needs.

The project demonstrates how collaboration, partnership, and shared ownership serve as critical mechanisms and platforms for intervention in complex systems. The POPSS initiative engaged a wide range of stakeholders across sectors, including healthcare providers, educators, community-based organizations, and public health practitioners. This multi-sector collaboration facilitated goal alignment, implemented interventions in unique settings, increased program reach, and set the stage for stakeholder investment in program sustainability.

Importantly, the initiative uses a staffing model in which academic partners provide technical and administrative infrastructure, while local community organizations lead program delivery and engagement efforts. This structure supported both fidelity to evidence-based programs and responsiveness to local context. The observed increase in partner engagement and dissemination channels over the first 2 years are indicators of sustainability. These findings align with prior research indicating that complex public health challenges require coordinated action across sectors and levels of influence (33, 40, 41).

By fostering shared ownership, CBSD approaches should enhance not only program uptake but also sustainability because they embed interventions within existing community structures. Additionally, local representation in program delivery strengthens relevance and acceptability among youth participants, thereby improving engagement and potential impact.

### Operationalizing systems thinking through iterative adaptation

This study also highlights the importance of continuous quality improvement (CQI) and iterative adaptation as core components of systems-based public health interventions. Through the use of Plan-Do-Study-Act cycles and recurring CBSD mapping activities, the POPSS initiative adapted to emerging challenges, evolving stakeholder priorities, and shifting community conditions over time. For example, transitions between evidence-based programs and modifications to implementation settings reflect the program’s capacity to adapt in response to real-time feedback and system conditions.

These adaptations reflect a central principle of systems thinking: interventions should remain responsive within dynamic and interconnected environments. Rather than viewing adaptation solely as a threat to implementation fidelity, this approach positions adaptation as a necessary process for sustaining relevance, strengthening contextual fit, and maximizing potential impact within complex systems. Integrating CQI processes within a CBSD framework may provide a practical model for balancing implementation rigor with the flexibility needed to support community-based public health interventions across diverse settings (35, 39).

### Implications for research, practice, and policy

The findings from this study have several important implications f or research, practice and policy. From a research perspective, this work contributes to the emerging evidence base on the application of CBSD approaches in adolescent SRH and offers a practical example for examining systems-level processes, stakeholder engagement and implementation outcomes within community settings.

From a practice perspective, the findings reinforce the importance of sustained community engagement, cross-sector collaboration, and adaptive implementation strategies when addressing complex adolescent health challenges. These approaches may strengthen local ownership, improve responsiveness to community needs, and support the implementation of multi-sector interventions in diverse settings.

From a policy perspective, these findings highlight the need for funding mechanisms, implementation supports, and evaluation frameworks that accommodate community-based, systems-oriented approaches. Policies that prioritize long-term collaboration, shared decision-making, and systems level capacity building may better support sustainable improvements in adolescent SRH outcomes than narrowly focused, single-intervention models.

### Limitations

Despite the strengths of a systems approach and community-based engagement, this study has several limitations. First, quantitative outcome data on adolescent sexual and reproductive health, such as pregnancy rates, STI incidence, or behavioral changes, have not yet been reported, limiting assessment of intervention impact. Stakeholder engagement relied on pre-existing networks, and no demographic information was collected in listening sessions, raising concerns about representativeness and potential selection bias. Additionally, our choice of evidence-based programs was constrained by funder requirements, which may have limited community-driven adaptation.

## Conclusion

Systems approaches and community-based system dynamics (CBSD) techniques offer promising strategies to address the complex, multi-level factors influencing adolescent SRH. Preliminary findings from the POPSS project suggest that engaging community stakeholders in locally driven CBSD mapping processes, fostering cross-sector collaboration and shared ownership, and embedding continuous quality improvement processes may strengthen the implementation and adaptation of multi-sector interventions over time.

By moving beyond isolated, individual-level prevention strategies and addressing the broader systems in which adolescents live, learn, and access health care, system-based initiatives may help reduce persistent barriers to health equity and improve access to evidence-based teen pregnancy prevention programming. Research is needed to understand how systems-level changes influence long-term adolescent health outcomes and to identify strategies to scale and sustain these approaches across diverse community settings.

## Data Availability

The original contributions presented in the study are included in the article/supplementary material, further inquiries can be directed to the corresponding author.
